# Noncollinear Edge Magnetism in Nanoribbons of Fe_3_GeTe_2_ and Fe_3_GaTe_2_

**DOI:** 10.1021/acs.nanolett.5c01890

**Published:** 2025-07-23

**Authors:** Ramon Cardias, Anders Bergman, Hugo U. R. Strand, R. B. Muniz, Marcio Costa

**Affiliations:** † Instituto de Física, 74350 Universidade Federal Fluminense, Niterói, Rio de Janeiro 24210-346, Brazil; ‡ Centro Brasileiro de Pesquisas Físicas (CBPF), Rua Dr Xavier Sigaud 150, Urca, Rio de Janeiro, Rio de Janeiro 22290-180, Brazil; § Department of Physics and Astronomy, 6233 Uppsala University, Box 516, SE-75120 Uppsala, Sweden; ∥ School of Science and Technology, 28110 Örebro University, SE-70182 Örebro, Sweden

**Keywords:** magnetism, field-free switching, 2D materials, spin dynamics, spin orbit torque, spintronics

## Abstract

Fe_3_GeTe_2_ and Fe_3_GaTe_2_ are ferromagnetic conducting materials of van der Waals type with unique magnetic properties that are highly promising for the development of new spintronic, orbitronic, and magnonic devices. Even in the form of two-dimensional-like ultrathin films, they exhibit a relatively high Curie temperature, magnetic anisotropy perpendicular to the atomic planes, and multiple types of Hall effects. We explore nanoribbons made from single layers of these materials and show that they display noncollinear magnetic ordering at their edges. This magnetic inhomogeneity allows angular momentum currents to generate magnetic torques at the sample edges, regardless of their polarization direction, significantly enhancing the effectiveness of magnetization manipulation in these systems. We also demonstrate that it is possible to rapidly reverse the magnetization direction of these nanostructures by means of spin–orbit and spin-transfer torques with rather low current densities, making them quite propitious for nonvolatile magnetic memory units.

## Graphical abstract

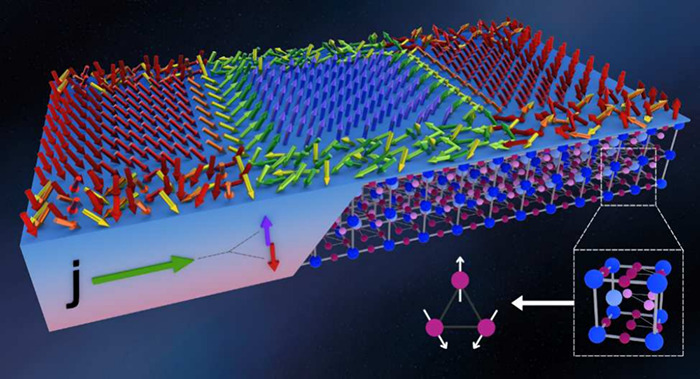

The ability to manipulate the magnetic ordering of nanoscale systems is of great interest to the magnetic industry. It enables the design and production of smaller and more efficient devices to process and store information. Spintronics, magnonics, and orbitronics have progressed greatly in recent years and are offering ingenious and innovative mechanisms to accomplish this, utilizing the transport of charge, spin, and orbital angular momentum across nanostructures.^1–4^

Two-dimensional (2D)-like materials, such as ultrathin films and multilayers, are currently receiving considerable attention due to their versatilities, particularly the magnetic van der Waals (vdW) ones,^5^ which can be exfoliated into ultrathin films and deposited with relative ease on different substrates, forming heterostructures with more comprehensive properties and functionalities. Among them, Fe_3_GeTe_2_ (FGeT) stands out as the first synthesized 2D metallic ferromagnet^6,7^ and is a very promising material for next-generation spintronic devices. It exhibits out-of-plane magnetic anisotropy, sizable spin–orbit interaction, and a relatively high anomalous Hall effect (AHE).^8,9^ Its Curie temperature (*T*_c_) may be controlled by doping,^10^ ionic gating,^6^ or through interaction with suitable substrates.

More recently, Fe_3_GaTe_2_ (FGaT) has also been synthesized with Ga atoms replacing Ge. The magnetic properties of the two materials are similar, but Fe_3_GaTe_2_ is even more attractive for practical use because its *T*_c_ value is above room temperature for thick films and remains rather large (≈240 K) for the monolayer.^11,12^

Different ways of manipulating the magnetic properties of nanostructured systems have been extensively investigated lately using current sources of distinct natures.^13–21^ A particularly promising strategy involves using the flow of angular momentum with a specific polarization to apply torque to local magnetic moments and excite spin dynamics within the magnetic system. Spin-polarized electric currents as well as pure spin- and orbital-angular momentum currents have been employed for such purposes. Spin-polarized electrical currents are typically generated by using magnetic fields or ferromagnetic (FM) materials as polarizers, while pure spin currents are most commonly produced by spin pumping or the spin Hall effect (SHE). These currents are usually generated in auxiliary materials and then injected to manipulate the magnetization of adjacent magnetic units. The effectiveness of the torque that they produce depends on both the direction and degree of spin polarization. Notably, both the spin-transfer torque (STT) and the spin–orbit torque (SOT) are zero if the spin-current polarization is parallel to the local magnetic moment.

Recently, significant investigations have been conducted into the production of orbital angular momentum currents and their role in driving spin dynamics. Although generating these currents through the orbital Hall effect does not require spin–orbit coupling (SOC) in the host material, the orbital torque (OT) depends on the spin–orbit interaction (SOI) within the magnetic unit. Both FGeT and FGaT exhibit sufficiently large SOC to generate SOT and OT, which may be used to control their magnetization. In addition to the relatively high anomalous Hall effect (AHE) observed in these materials,^11,22^ an electric current flowing through them may also give rise to the conventional (SHE) and spin anomalous Hall effect (sAHE)^23^ due to SOI.

Both systems crystallize in a hexagonal structure, belonging to crystallographic point group *D*_6*h*_ of space group *P*6_3_/*mmc* (no. 194). It consists of layers stacked along the *c* axis, each of which comprises an Fe_3_Ge­(Ga) sublayer sandwiched between two atomic planes of Te. A single layer of Fe_3_Ge­(Ga)­Te_2_ contains five atomic planes and belongs to point group symmetry *D*_3*h*_. Figure 1a illustrates its lattice structure and unit cell, highlighting the two non-equivalent Fe atoms that occupy octahedral (Fe_I_) and tetrahedral (Fe_II_) sites, respectively.

**Figure 1:**
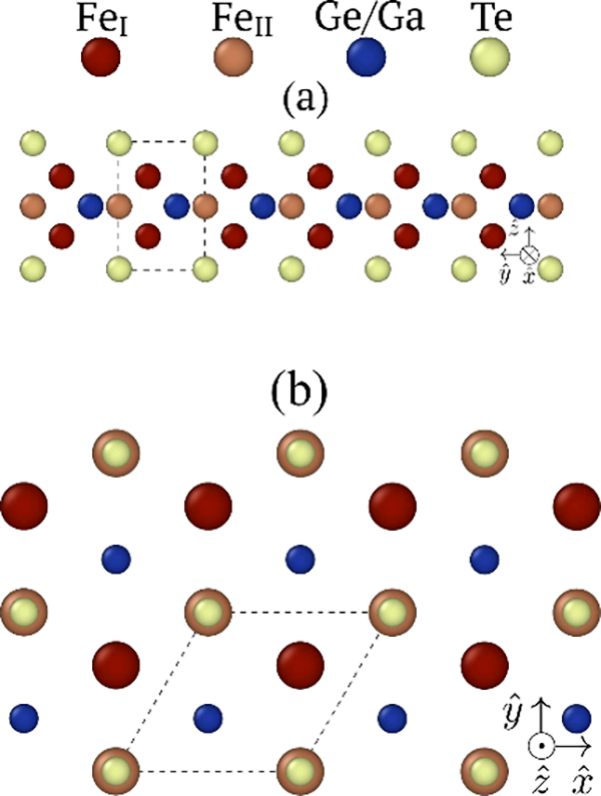
Schematic representation of the crystalline structure of a monolayer of Fe_3_Ge­(Ga)­Te_2_: (a) side view and (b) top view. The dashed lines portray the unit cell boundaries.

Here, we investigate nanoribbons made from single layers of FGeT and FGaT with armchair-shaped edges. The presence of these borders reduces the *D*_3*h*_ symmetry of the monolayer, giving rise to a complex competition between the bilinear exchange and the Dzyaloshinskii–Moriya (DM) interactions, producing a ground state with noncollinear magnetic ordering at the edges. This feature facilitates the manipulation of magnetization of these systems through angular momentum torques, as the edges experience torque regardless of the angular momentum polarization direction.

We shall start with density functional theory (DFT)^200,201^ band structure calculations for single layers of FGeT and FGaT, using the plane-wave-based code Quantum ESPRESSO.^202^ The results are shown in the Supporting Information alongside our band calculations using pseudo-atomic orbitals with the PAOFLOW code.^24^ Clearly, the two sets are in excellent agreement. Both materials are clearly metallic and exhibit FM ground states with perpendicular magnetic anisotropy. The calculated magnetic moments of Fe_I_ (Fe_II_) are 2.64 (1.47) μ_B_ for FGeT and 2.48 (1.48) μ_B_ for FGaT, in good agreement with previous studies.^25,26^ We also calculated the single-ion magnetic anisotropy energy (MAE) per Fe atom, obtaining values of 1.02 meV for FGeT and 0.83 meV for FGaT, which are consistent with the results reported in ref 25.

The energy bands calculated using DFT for nanoribbons with armchair edges extracted from the monolayer of Fe_3_GeTe_2_ and Fe_3_GaTe_2_ are shown in Figure S2 of the Supporting Information. The metallic nature of the nanoribbons is evidenced by electronic states that cross the Fermi level. The color coding scheme illustrates the spatial character of the corresponding eigenstates, where red denotes high probability amplitudes at the edge sites and blue represents high probability amplitudes in the central region of the ribbon. Both ribbons are approximately 18 Å in breadth.

A useful strategy for exploring the ground state spin configuration of these magnetic systems is to represent them by an effective spin Hamiltonian as follows:

1 $$H=-\underset{i,j}{\sum }J_{ij}{\mathrm{m̂}}_{i}\cdot {\mathrm{m̂}}_{j}-\underset{i,j}{\sum }{\mathrm{D⃗}}_{ij}\cdot \left({\mathrm{m̂}}_{i}\times {\mathrm{m̂}}_{j}\right)-K\underset{i}{\sum }{\left({\mathrm{m̂}}_{i}\cdot {{\mathrm{m̂}}_{i}}^{k}\right)}^{2}$$

where *J*_*ij*_ represents the pairwise effective exchange interaction between local magnetic moments *m⃗*_*i*_ = *m*_*i*_ *m̂*_*i*_ and *m⃗*_*j*_ = *m*_*j*_ *m̂*_*j*_ located at sites *i* and *j*, respectively. Here, *m̂*_*i*_ symbolizes the unit vector along the local moment direction, and *m*_*i*_ is its magnitude. *D⃗*_*ij*_ denotes the DM vector, associated with the effective DM interaction due to SOC. The last term typifies the single-ion anisotropy with intensity proportional to K , and *m̂*_*i*_^*k*^ indicates the unit vector along the direction of the uniaxial anisotropy. The magnitudes of the moments were incorporated into the interaction constants, which are expressed in energy units.

The effective spin interactions *J*_*ij*_, *D⃗*_*ij*_, and K can be completely determined from electronic structure calculations, as briefly described in Sections S4 and S5 of the Supporting Information. Here, we use the Liechtenstein, Katsnelson, Antropov, and Gubanov (LKAG) and Green’s function formalisms^27–30^ to compute *J*_*ij*_ and *D⃗*_*ij*_ for any pair of Fe magnetic moments. Green’s functions involved are expanded in terms of Chebyshev polynomials using the kernel polynomial method,^31^ to improve computational efficiency.

Figure 1 shows that the Fe atoms in a single layer of FeGe­(Ga)­T are arranged in three atomic planes. The one in the middle of the layer contains only Fe_II_ and Ge (Ga) atoms and is sandwiched by two others, formed solely by Fe_I_ atoms. It is instructive to calculate the values of *J*_*ij*_ and *D⃗*_*ij*_ for the single layer as a reference for later comparison to those of the nanoribbons. The results are displayed in Figure S3 as functions of the interatomic distances, highlighting the inter- and in-plane pairs of Fe atoms. They are in good agreement with those reported in refs 25 and 26 . It is worth mentioning that the minimization of the total energy, using eq 1 along with the values of *J*_*ij*_ and K for the single layer, confirms the FM configuration of the ground state with magnetic moments oriented perpendicular to the layer, consistent with DFT calculations (the contribution to the total energy coming from the Dzyaloshinskii–Moriya interaction vanishes in the single layer due to symmetry). Using the calculated values of these parameters, we have also performed spin dynamic simulations to determine the critical temperatures *T*_c_ of single layers of FGeT and FGaT. We found *T*_c_ = 280 K for FGeT and *T*_c_ = 420 K for FGaT, which are in good agreement with both theoretical predictions and experimental results.^12,32^ Additional details are provided in the Supporting Information.

The same formalism has been employed to calculate the values of *J*_*ij*_ and *D⃗*_*ij*_ for FGeT and FGaT nanoribbons with armchair borders. Far from the edges, the results are very similar to those for the single layer, as expected. However, near them, the differences are significant. Hence, to simplify the presentation, we have selected an Fe atom located at one of the edges and illustrated its interaction with neighboring Fe atoms in Figure 2 (here, we note that the borders of the armchair-edged ribbons are equivalent due to mirror symmetry $M_{x}$ or $\tau M_{x}$ ). We note that the highest value of *J*_*ij*_ is the interplane FM coupling between the nearest-neighbor Fe_I_ atoms, as Figure 2 illustrates. Next, we have interplanar couplings between Fe_I_ and its nearest-neighbor Fe_II_ atoms, which exhibit both FM and antiferromagnetic (AFM) contributions. In particular, the in-plane couplings between the next-nearest Fe_I_ atoms, which are AFM in the case of FGeT, also display both FM and AFM contributions in FGaT. Slight variations in the interatomic distances between originally equidistant Fe atoms may occur because of spatial relaxations considered in our DFT calculations. The *J*_*ij*_ values between these nearly equidistant Fe atoms can differ significantly, as they become non-equivalent due to the breaking of translation symmetry along the transverse direction of the nanoribbon. It is noteworthy that orbital contributions to the effective exchange interactions *J*_*ij*_ between Fe atoms can have opposite signs, leading to a net coupling that may be either AFM or FM.^33^ Relaxations influence these orbital contributions and can alter the sign of *J*_*ij*_, as illustrated in Figure 2. These effects are absent in the pristine monolayer. The significant changes in *D⃗*_*ij*_ caused by the loss of translational symmetry in the transverse direction of the nanoribbons are illustrated in Figure S4.

**Figure 2:**
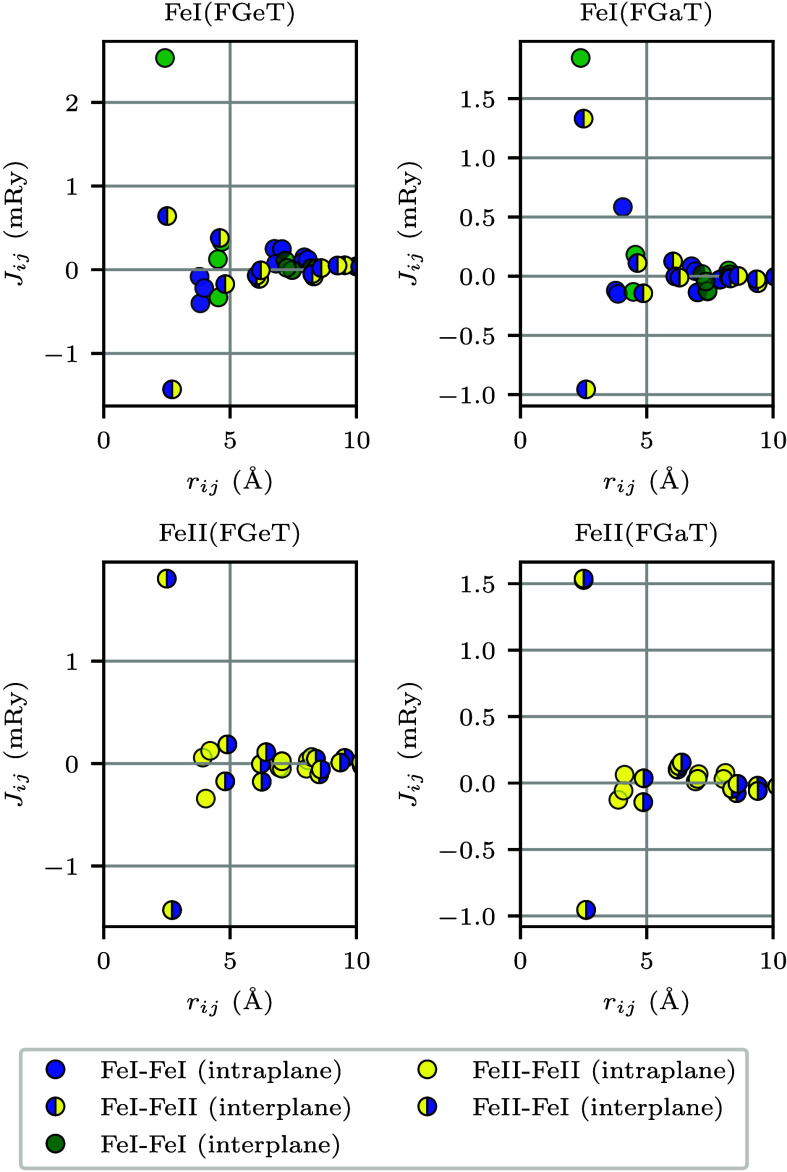
Effective exchange couplings between the magnetic moment of an Fe atom (Fe_I_, top panels; Fe_II_, bottom panels) located at the edge of an armchair nanoribbon and its neighboring Fe atoms, calculated as functions of their interatomic distances. Solid circles represent interactions between Fe atoms of the same type: (blue) Fe_I_–Fe_I_ in-plane, (green) Fe_I_–Fe_I_ interplane, and (yellow) Fe_II_–Fe_II_ in-plane. Interplane interactions between Fe atoms of distinct types are depicted by circles with two colors: (blue left/yellow right) Fe_I_–Fe_II_ and (yellow left/blue right) Fe_II_–Fe_I_.

These parameters have been employed to determine the magnetic configuration in the ground state of the nanoribbons by using the effective spin Hamiltonian given in eq 1, following the methodology of the UppASD code.^203^ The results are illustrated in Figure 3 and clearly reveal the noncollinear ground-state spin arrangements at their edges. This feature greatly expands the possibilities of manipulating the magnetization of nanostructures of these materials by means of angular-momentum torques because they become effective regardless of the angular-momentum polarization direction.

**Figure 3:**
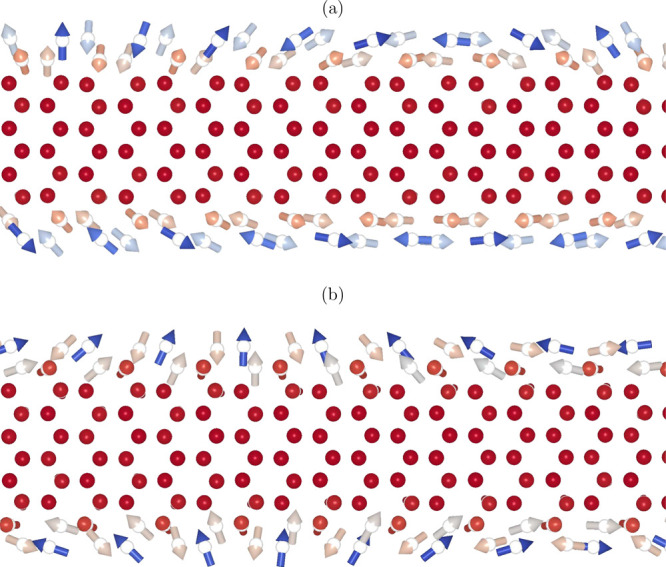
Ground-state spin configurations of armchair-edged nanoribbons made from single layers of (a) FGaT and (b) FGeT, both with approximately 18 Å in breadth.

It is also worth mentioning that when an electron hops between atoms with noncollinear magnetic moments, it generally acquires a Berry phase, which acts as a fictitious external magnetic field. This is an important ingredient in engineering *p*-wave superconductors^34^ and also one of the mechanisms behind the existence of the spin Hall effect in the absence of spin–orbit coupling.^35^ Recently, a long-range Josephson supercurrent has been observed flowing across a flake of FGeT that connects two spin-singlet superconductors composed of NbSe_2_.^36^ A singlet spin supercurrent is predicted to decay rapidly upon entering a FM, such as FGeT. However, it has been observed that the supercurrent survived for much longer distances than expected and exhibits higher density at the flake edges. Our results support the hypothesized presence of noncollinear magnetism in the FGeT flake that could promote electronic transitions to a spin-triplet state, thereby reducing the supercurrent damping, particularly at the edges.

The possibility of field-free switching of magnetic states in vdW magnets is very interesting from a technological standpoint, and a recent study has indicated the possibility of current-induced switching of FGaT flakes deposited on Pt.^37^ In addition, the noncollinear antiferromagnet Mn_3_Sn has shown potential for efficient SOT-driven field-free switching owing to its strong magnetic spin Hall effect (mSHE).^38^ The mSHE is the odd part of the full conductivity tensor. The non-negligible terms will depend not only on the crystal symmetry but also on the magnetic configuration. In the case of Mn_3_Sn, for a given magnetic configuration (see the Table of Content (ToC) graphic), both σ_*xz*_^*y*^ and σ_*xz*_^*z*^ are finite. This means that, when a current density is injected in the *x* direction, a transverse spin-polarized current is generated in the *z* direction toward the interface between Mn_3_Sn and FGe­(Ga)­T, with polarizations along the *y* and *z* axes. Here, we investigate how the magnetic states of FGaT and FGeT nanoribbons can be controlled by current-induced spin torques, assuming that they are placed atop Mn_3_Sn. In the simulations, we focus on the effect of SOT with the assumption that the dominant contribution in these systems comes from the mSHE^39^ of the Mn_3_Sn substrate. How to incorporate these torques into an ASD framework has been explained in detail by Meo et al.^40^ The strength and effectiveness of the SOT are determined by the current density *j⃗*, the spin Hall angle θ_SH_, and the Gilbert damping parameter α. Further details on the SOT treatment in our simulations are given in the Supporting Information. The value of θ_SH_ = 0.3 is taken from the experimental data reported in ref 38 to emulate the situation in which the nanoribbons are deposited on Mn_3_Sn. In our simulations, we assume that the electric current flows primarily through the Mn_3_Sn substrate, with the effects of electrical conduction in the FGe­(Ga)­T nanoribbon considered negligible.

In Figure 4, we show the magnetic response of FGaT and FGeT nanoribbons when excited with a current density *j* = 2 × 10^12^ A/m^2^ and a polarization opposite their initial magnetization. The temperature of the systems is fixed to *T* = 1.0 K. For both nanoribbons, we observe a switching on a time scale of less than 100 ps, showing the feasibility of rapid manipulation of these spin states by reasonable current densities. Videos illustrating the dynamics of magnetization switching, derived from our simulations for both nanoribbons, are available in the Supporting Information.

**Figure 4:**
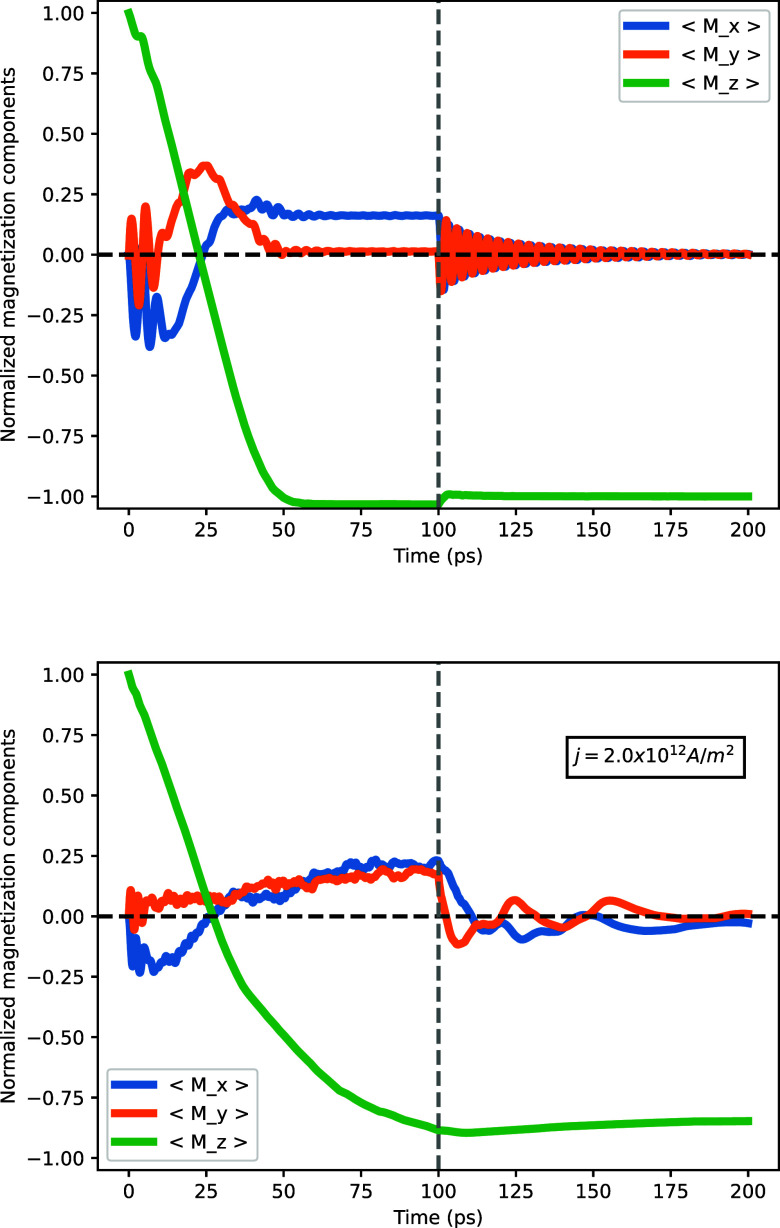
Average projected magnetization of the FGaT (top) and FGeT (bottom) nanoribbons during the spin dynamics process. During the first 200 ps, we show the magnetization switch dynamics via spin–orbit torque (SOT), and in the last 100 ps (after the vertical dashed line), the system is allowed to relax. The current applied is *j* = 2 × 10^12^ A/m^2^ and θ_SH_ = 0.3.

Figure 4 also illustrates what happens when the excitation current in our simulations is turned off some time after the magnetization has been switched. At *t* = 200 ps, a dashed vertical line marks this moment. From this point onward, we observe that the expectation value of *M*_*z*_ remains stable, while ⟨*M*_*x*_⟩ and ⟨*M*_*y*_⟩ gradually relax to zero.

In summary, we have demonstrated that nanoribbons of Fe_3_GeTe_2_ and Fe_3_GaTe_2_ exhibit unique noncollinear edge magnetic configurations, arising from a balance between exchange and Dzyaloshinskii–Moriya interactions at the edges, which are much different from the monolayer interactions. This feature not only enables effective magnetization manipulation via spin-transfer and spin–orbit torques but also allows for rapid magnetization switching at significantly lower current densities compared to conventional FM systems. These findings emphasize the promising applications of Fe_3_GeTe_2_ and Fe_3_GaTe_2_ in spintronic, orbitronic, and magnonic devices, offering solutions for nonvolatile memory and high-speed data processing. Moreover, the realization of noncollinear magnetism at the edges also sheds light in previous experiments and offers a set of new possibilities to be explored further. Our theoretical predictions could be experimentally validated using a combination of high-resolution magnetic imaging and transport techniques. For example, spin-polarized scanning tunneling microscopy (SP-STM) could directly image the predicted edge noncollinear magnetic configurations at the nanoscale. Together, our results establish these materials as versatile platforms for advancing the frontiers of low-dimensional magnetism and device miniaturization.

## Supplementary Material







## Notes

Video of our simulations illustrating the influence of magnetic edge noncollinearity on the magnetization switching process in this nanostructure (MP4)
Video of our simulations illustrating the influence of magnetic edge noncollinearity on the magnetization switching process in this nanostructure (MP4)
Additional details of our electronic DFT calculations, including the energy band results for single layers and nanoribbons of Fe_3_GeTe_2_ and Fe_3_GaTe_2_, more information on the PAO projection method and the Hall conductivity calculations for single layers of Fe_3_GeTe_2_ and Fe_3_GaTe_2_, and details regarding the effective spin Hamiltonian used in our simulations for our atomistic spin dynamics simulations, including the obtention of the effective interactions involved (PDF)

## References

[1] Sinova J., Valenzuela S. O., Wunderlich J., Back C., Jungwirth T. (2015). Spin Hall
effects. Rev. Mod. Phys..

[2] Jo D., Go D., Choi G.-M., Lee H.-W. (2024). Spintronics meets orbitronics: Emergence
of orbital angular momentum in solids. npj Spintronics.

[3] Ralph D. C., Stiles M. D. (2008). Spin transfer torques. J. Magn.
Magn. Mater..

[4] Hals K. M. D., Brataas A. (2013). Phenomenology
of current-induced spin-orbit torques. Phys.
Rev. B.

[5] Wang Q. H. (2022). The Magnetic Genome
of Two-Dimensional van der Waals Materials. ACS Nano.

[6] Deng Y., Yu Y., Song Y., Zhang J., Wang N. Z., Sun Z., Yi Y., Wu Y. Z., Wu S., Zhu J., Wang J., Chen X. H., Zhang Y. (2018). Gate-tunable room-temperature ferromagnetism
in two-dimensional Fe_3_GeTe_2_. Nature.

[7] Fei Z., Huang B., Malinowski P., Wang W., Song T., Sanchez J., Yao W., Xiao D., Zhu X., May A. F., Wu W., Cobden D. H., Chu J.-H., Xu X. (2018). Two-dimensional itinerant ferromagnetism in atomically thin Fe_3_GeTe_2_. Nat. Mater..

[8] Wang Y., Xian C., Wang J., Liu B., Ling L., Zhang L., Cao L., Qu Z., Xiong Y. (2017). Anisotropic
anomalous Hall effect in triangular itinerant ferromagnet Fe_3_GeTe_2_. Phys. Rev. B.

[9] Kim K. (2018). Large anomalous Hall
current induced by topological nodal lines in
a ferromagnetic van der Waals semimetal. Nat.
Mater..

[10] Jang S. W., Yoon H., Jeong M. Y., Ryee S., Kim H.-S., Han M. J. (2020). Origin of ferromagnetism and the effect of doping on
Fe_3_GeTe_2_. Nanoscale.

[11] Zhang G., Guo F., Wu H., Wen X., Yang L., Jin W., Zhang W., Chang H. (2022). Above-room-temperature
strong intrinsic
ferromagnetism in 2D van der Waals Fe_3_GaTe_2_ with
large perpendicular magnetic anisotropy. Nat.
Commun..

[12] Wang M., Lei B., Zhu K., Deng Y., Tian M., Xiang Z., Wu T., Chen X. (2024). Hard ferromagnetism in van der Waals Fe_3_GaTe_2_ nanoflake down to monolayer. npj 2D Materials
and Applications.

[13] Miron I. M., Garello K., Gaudin G., Zermatten P.-J., Costache M. V., Auffret S., Bandiera S., Rodmacq B., Schuhl A., Gambardella P. (2011). Perpendicular
switching of a single
ferromagnetic layer induced by in-plane current injection. Nature.

[14] Zhang K., Han S., Lee Y., Coak M. J., Kim J., Hwang I., Son S., Shin J., Lim M., Jo D., Kim K., Kim D., Lee H.-W., Park J.-G. (2021). Gigantic Current Control of Coercive
Field and Magnetic Memory Based on Nanometer-Thin Ferromagnetic van
der Waals Fe_3_GeTe_2_. Adv.
Mater..

[15] Zhang K., Lee Y., Coak M. J., Kim J., Son S., Hwang I., Ko D.-S., Oh Y., Jeon I., Kim D., Zeng C., Lee H.-W., Park J.-G. (2021). Highly Efficient
Nonvolatile Magnetization Switching and Multi-Level States by Current
in Single Van der Waals Topological Ferromagnet Fe_3_GeTe_2_. Adv. Funct. Mater..

[16] Wang H. (2023). Room temperature energy-efficient
spin-orbit torque switching in
two-dimensional van der Waals Fe_3_GeTe_2_ induced
by topological insulators. Nat. Commun..

[17] Yan S., Tian S., Fu Y., Meng F., Li Z., Lei H., Wang S., Zhang X. (2024). Highly Efficient Room-Temperature
Nonvolatile Magnetic Switching by Current in Fe_3_GaTe_2_ Thin Flakes. Small.

[18] Alghamdi M., Lohmann M., Li J., Jothi P. R., Shao Q., Aldosary M., Su T., Fokwa B. P. T., Shi J. (2019). Highly Efficient
Spin-Orbit Torque and Switching of Layered Ferromagnet Fe_3_GeTe_2_. Nano Lett..

[19] Kajale S. N., Nguyen T., Chao C. A., Bono D. C., Boonkird A., Li M., Sarkar D. (2024). Current-induced
switching of a van der Waals ferromagnet
at room temperature. Nat. Commun..

[20] Dai Y. (2024). Interfacial magnetic
spin Hall effect in van der Waals Fe_3_GeTe_2_/MoTe_2_ heterostructure. Nat. Commun..

[21] Zhou J., Charlier J.-C. (2021). Controllable spin
current in van der Waals ferromagnet
Fe_3_GeTe_2_. Physical Review
Research.

[22] Lin X., Ni J. (2019). Layer-dependent intrinsic anomalous Hall effect in Fe_3_GeTe_2_. Phys. Rev. B.

[23] Iihama S., Taniguchi T., Yakushiji K., Fukushima A., Shiota Y., Tsunegi S., Hiramatsu R., Yuasa S., Suzuki Y., Kubota H. (2018). Spin-transfer
torque
induced by the spin anomalous Hall effect. Nature
Electronics.

[200] Hohenberg P., Kohn W. (1964). Inhomogeneous Electron Gas. Phys. Rev..

[201] Kohn W., Sham L. J. (1965). Self-Consistent
Equations Including
Exchange and Correlation Effects. Phs. Rev..

[202] Giannozzi P. (2017). Advanced capabilities for materials
modelling with
Q uantum ESPRESSO. J. Phys.: Condens. Matter.

[24] Buongiorno
Nardelli M., Cerasoli F. T., Costa M., Curtarolo S., De Gennaro R., Fornari M., Liyanage L., Supka A. R., Wang H. (2018). PAOFLOW: A utility to construct and operate on *ab initio* Hamiltonians from the projections of electronic wavefunctions on
atomic orbital bases, including characterization of topological materials. Comput. Mater. Sci..

[25] Ghosh S., Ershadrad S., Borisov V., Sanyal B. (2023). Unraveling effects
of electron correlation in two-dimensional Fe_
*n*
_GeTe_2_ (*n* = 3, 4, 5) by dynamical
mean field theory. npj Computational Materials.

[26] Ruiz A. M., Esteras D. L., López-Alcalá D., Baldoví J. J. (2024). On the
Origin of the Above-Room-Temperature Magnetism in the 2D van der Waals
Ferromagnet Fe_3_GaTe_2_. Nano Lett..

[27] Szilva A., Kvashnin Y., Stepanov E. A., Nordström L., Eriksson O., Lichtenstein A. I., Katsnelson M. I. (2023). Quantitative
theory of magnetic interactions in solids. Rev.
Mod. Phys..

[28] Liechtenstein A. I., Katsnelson M. I., Antropov V. P., Gubanov V. A. (1987). Local spin density
functional approach to the theory of exchange interactions in ferromagnetic
metals and alloys. J. Magn. Magn. Mater..

[29] Cardias R., Szilva A., Bezerra-Neto M. M., Ribeiro M. S., Bergman A., Kvashnin Y. O., Fransson J., Klautau A. B., Eriksson O., Nordström L. (2020). First-principles
Dzyaloshinskii-Moriya interaction
in a non-collinear framework. Sci. Rep..

[30] Frota-Pessôa S., Muniz R. B., Kudrnovský J. (2000). Exchange coupling
in transition-metal
ferromagnets. Phys. Rev. B.

[31] Weiße A., Wellein G., Alvermann A., Fehske H. (2006). The kernel polynomial
method. Rev. Mod. Phys..

[32] Tan C., Lee J., Jung S.-G., Park T., Albarakati S., Partridge J., Field M. R., McCulloch D. G., Wang L., Lee C. (2018). Hard magnetic
properties in nanoflake
van der Waals Fe_3_GeTe_2_. Nat. Commun..

[33] Kvashnin Y., Cardias R., Szilva A., Di Marco I., Katsnelson M., Lichtenstein A., Nordström L., Klautau A., Eriksson O. (2016). Microscopic
Origin of Heisenberg and Non-Heisenberg Exchange Interactions in Ferromagnetic
bcc Fe. Phys. Rev. Lett..

[34] Chatterjee P., Banik S., Bera S., Ghosh A. K., Pradhan S., Saha A., Nandy A. K. (2024). Topological
superconductivity by
engineering noncollinear magnetism in magnet/superconductor heterostructures:
A realistic prescription for the two-dimensional Kitaev model. Phys. Rev. B.

[35] Zhang Y., Železný J., Sun Y., van den
Brink J., Yan B. (2018). Spin Hall effect emerging from a
noncollinear magnetic lattice without spin-orbit coupling. New J. Phys..

[36] Hu G., Wang C., Wang S., Zhang Y., Feng Y., Wang Z., Niu Q., Zhang Z., Xiang B. (2023). Long-range
skin Josephson supercurrent across a van der Waals ferromagnet. Nat. Commun..

[203] Eriksson, O.; Bergman, A.; Bergqvist, L.; Hellsvik, J. Atomistic Spin Dynamics: Foundations and Applications. Oxford University Press: 2017.

[37] Yun C. (2023). Efficient current-induced spin torques and field-free magnetization
switching in a room-temperature van der Waals magnet. Science Advances.

[38] Hu S., Shao D.-F., Yang H., Pan C., Fu Z., Tang M., Yang Y., Fan W., Zhou S., Tsymbal E. Y., Qiu X. (2022). Efficient perpendicular magnetization
switching by a magnetic spin Hall effect in a noncollinear antiferromagnet. Nat. Commun..

[39] Manchon A., Železný J., Miron I., Jungwirth T., Sinova J., Thiaville A., Garello K., Gambardella P. (2019). Current-induced
spin-orbit torques in ferromagnetic and antiferromagnetic systems. Rev. Mod. Phys..

[40] Meo A., Cronshaw C. E., Jenkins S., Lees A., Evans R. F. L. (2023). Spin-transfer
and spin-orbit torques in the Landau-Lifshitz-Gilbert equation. J. Phys.: Condens. Matter.

