# Supplementary material for: Noncollinear Edge Magnetism in Nanoribbons of Fe3GeTe2 and Fe3GaTe2
Source: Nano Lett. 2025 Jul 23;25(31):11797–802. doi: 10.1021/acs.nanolett.5c01890 (PMC12333401; doi:10.1021/acs.nanolett.5c01890)
Supplement: Supplementary file 3 [file nl5c01890_si_003.pdf]

Non-collinear edge magnetism in nanoribbons of Fe_3GeTe_2 and Fe_3GaTe_2: Supporting Information

Ramon Cardias,[1,2,*] Anders Bergman,[3] Hugo U. R. Strand,[4] R. B. Muniz,[1] and Marcio Costa[1]

[1] *Instituto de Física, Universidade Federal Fluminense, 24210-346, Niterói RJ, Brazil*
[2] *Centro Brasileiro de Pesquisas Físicas (CBPF), Rua Dr Xavier Sigaud 150, Urca, 22290-180, Rio de Janeiro-RJ, Brazil*
[3] *Department of Physics and Astronomy, Uppsala University, Box 516, SE-75120 Uppsala, Sweden*
[4] *School of Science and Technology, Örebro University, SE-70182 Örebro, Sweden*

(Dated: May 30, 2025)

S1: DENSITY FUNCTIONAL THEORY CALCULATIONS

We performed Density Functional Theory (DFT) calculations using the plane-wave code Quantum Espresso. Exchange-correlation (XC) effects were treated within the generalized gradient approximation (GGA) using the Perdew-Burke-Ernzerhof (PBE) functional [1]. Ionic potentials were described using projector augmented-wave (PAW) potentials [2] sourced from the pslibrary database [3]. The wavefunction cutoff energy was set to 60 Rydbergs, while the charge density cutoff was 10 times larger. Reciprocal space sampling was performed with a 15×15×1 grid for the single layers and a 1×9×1 grid for the nanoribbons. The magnetic anisotropy energy (MAE) was calculated with a 49×49×1 grid to ensure proper convergency. To eliminate spurious interactions, a 15 Å vacuum was introduced in all non-periodic directions. The calculated lattice parameters for single layers of Fe_3GeTe_2 and Fe_3GaTe_2 are 4.07 Å and 4.06 Å, respectively, which agree with previously reported values [4, 5]. Structural optimization was carried out until the Hellmann–Feynman force values fell below 0.01 eV/Å

S2: THE PSEUDO ATOMIC ORBITAL PROJECTION METHOD

Plane-wave (PW) bases are widely employed in DFT calculations due to their versatility and accuracy. However, when addressing local representations, the use of a PW basis can pose certain challenges. For example, the LKAG equations require local propagators, making PWs unsuitable for such purposes. To construct these local representations, we utilize the pseudo-atomic orbital (PAO) method as implemented in the paoflow code [6–8]. The PAO method involves projecting Kohn-Sham orbitals, which are represented by thousands of PWs, onto an atomic orbital basis. We have utilized the pseudo-atomic basis built into the pseudopotentials used for the DFT calculations, which includes nine orbitals (the five $3d$ and the $4sp$ complex) for all atoms.

The atomic spin-orbit coupling (SOC) is introduced in the PAO Hamiltonian via an effective approximation,

$$H_{\mathrm{SOC}} = \sum_i \sum_{\mu\nu} \sum_{\sigma\sigma'} \xi_i^{\mu\nu} \langle i\mu\sigma | L \cdot S | i\nu\sigma' \rangle c^\dagger_{i\mu\sigma} c_{i\nu\sigma'}, \quad (1)$$

where the operator $c^\dagger_{i\mu\sigma}(c_{i\mu\sigma})$ creates (annihilates) an electron with spin projection σ at the atomic site i and orbital μ. The L and S denote the orbital and spin angular momentum operators, respectively. The SOC strength, $\xi_i^{\mu\nu}$, is determined by fitting to fully relativistic DFT calculations. This methodology has been successfully applied in studies of other topological and magnetic systems [9, 10]. The SOC coupling strengths are: $\xi^d_{\mathrm{Fe}} = 40$ meV, $\xi^p_{\mathrm{Ge}} = 200$ meV, $\xi^p_{\mathrm{Ga}} = 150$ meV, and $\xi^p_{\mathrm{Te}} = 600$ meV. Figure 1 compares the energy bands of Fe_3GeTe_2 and Fe_3GaTe_2 monolayers calculated using DFT and the PAO Hamiltonian. The results from both methods exhibit excellent agreement.

Using linear response theory and the PAO Hamiltonian, we have also computed the anomalous Hall (AH), spin Hall (SH), and orbital Hall (OH) conductivities for both materials. The calculations were performed on the basis of the following expressions:

$$\sigma^\eta_{xy} = \frac{e}{(2\pi)^2} \sum_n \int_{BZ} d^2k f_{n\mathbf{k}}\, \Omega^{X_\eta}_{n,\mathbf{k}}, \quad (2)$$

where $\eta = 0$ or z. Here, σ^0_{xy} represents the anomalous Hall conductivity, and $\eta = z$ embodies the spin Hall $\sigma^{z(\mathrm{S})}_{xy}$ and orbital Hall $\sigma^{z(\mathrm{O})}_{xy}$ conductivities with angular momentum polarization along the $\hat{z}$ direction. The Berry curvatures

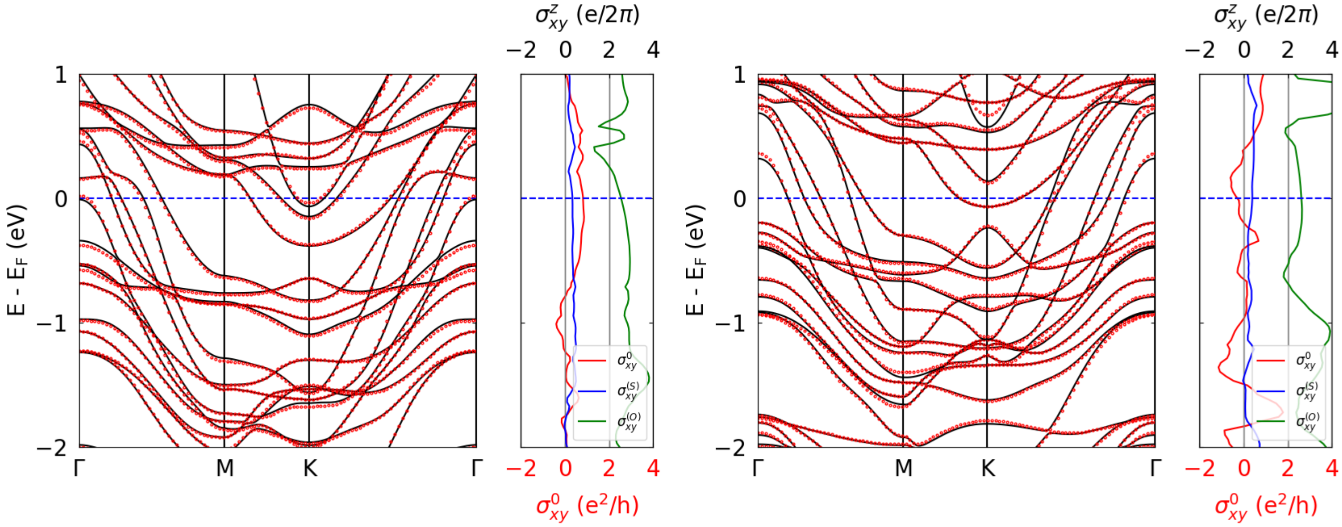

FIG. 1: Energy band structures of single layers of FGeT (a) and FGaT (b), calculated along some high symmetry directions of the two-dimensional Brillouin zone. The black solid lines represent the fully relativistic DFT calculations and the red dotted lines the PAOFLOW with ad-hoc SOC. The SOC coupling strengths are : $\xi^d_{Fe} = 40$ meV, $\xi^p_{Ge} = 200$ meV, $\xi^p_{Ga} = 150$ meV, $\xi^p_{Te} = 600$ meV. The Fermi energy is depicted by the blue horizontal dashed line. The associated side panels show the anomalous Hall conductivity σ^0_{xy}, together with the spin Hall $\sigma^{z(\mathrm{S})}_{xy}$ and orbital Hall $\sigma^{z(\mathrm{O})}_{xy}$ conductivities, with polarization ($\hat{z}$) perpendicular to the layers, calculated as functions of energy for (a) FGeT and (b) FGaT.

Monolayer	$\mathrm{m}_{\mathrm{Fe}_{\mathrm{I}}}$ (μ_{B})	$\mathrm{m}_{\mathrm{Fe}_{\mathrm{II}}}$ (μ_{B})	$\mathcal{K}$ (meV/ Fe atom)
FGeT	2.63	1.47	1.02
FGaT	2.48	1.48	0.83

TABLE I: Magnetic moments of $\mathrm{Fe_I}$ and $\mathrm{Fe_{II}}$ atoms in single layers of $\mathrm{Fe_3GeTe_2}$ and $\mathrm{Fe_3GaTe_2}$, together with the calculated magnetic anisotropy constants for both materials.

$\Omega^{X_\eta}_{n,\mathbf{k}}$ are given by

$$\Omega^{X_\eta}_{n,\mathbf{k}} = 2\hbar \sum_{m\neq n} \mathrm{Im}\left[\frac{\langle u_{n,\mathbf{k}}|j^{X_\eta}_{y,\mathbf{k}}|u_{m,\mathbf{k}}\rangle\langle u_{m,\mathbf{k}}|v_x(\mathbf{k})|u_{n,\mathbf{k}}\rangle}{(E_{n,\mathbf{k}} - E_{m,\mathbf{k}} + i0^+)^2}\right], \tag{3}$$

where the velocity operators are given by $v_{x(y)}(\mathbf{k}) = \hbar^{-1}\partial\mathcal{H}_{\mathrm{PAO}}(\mathbf{k})/\partial k_{x(y)}$, and $|u_{n(m),\mathbf{k}}\rangle$ is the periodic part of Bloch wave function with energy $E_{n(m),\mathbf{k}}$. The component of the current density operator along the $\hat{y}$ direction is defined by $j^{X_\eta}_{y,\mathbf{k}} = (X_\eta v_y(\mathbf{k}) + v_y(\mathbf{k})X_\eta)/2$, where $X_0 = \hat{\mathbb{1}}$ and $X_z = \hat{s}_z(\hat{\ell}_z)$ is the z-component of the spin (orbital) angular momentum operator. Here, we employ the intra-atomic approximation for the OAM operator, which offers a satisfactory description of the OHE in itinerant ferromagnets [11–14]. Contributions beyond the intra-atomic approximation have been addressed in the references 15–17.

The side panels in Fig.1 show the results of σ^0_{xy}, $\sigma^{z(\mathrm{S})}_{xy}$ and $\sigma^{z(\mathrm{O})}_{xy}$, calculated as functions of energy for single layers of both $\mathrm{Fe_3GeTe_2}$ and $\mathrm{Fe_3GaTe_2}$. Table I shows the DFT calculated values of the magnetic moments of $\mathrm{Fe_I}$ and $\mathrm{Fe_{II}}$ atoms, as well as the magnetic anisotropy (MAE) constants for single layers of $\mathrm{Fe_3GeTe_2}$ and $\mathrm{Fe_3GaTe_2}$. The MAE was calculated via total energy difference from a fully relativistic DFT calculation, MAE = $\mathrm{E}_{(100)} - \mathrm{E}_{(001)}$. Positive values favor an out-of-plane easy axis. Our values are in good agreement with the literature [5, 18].

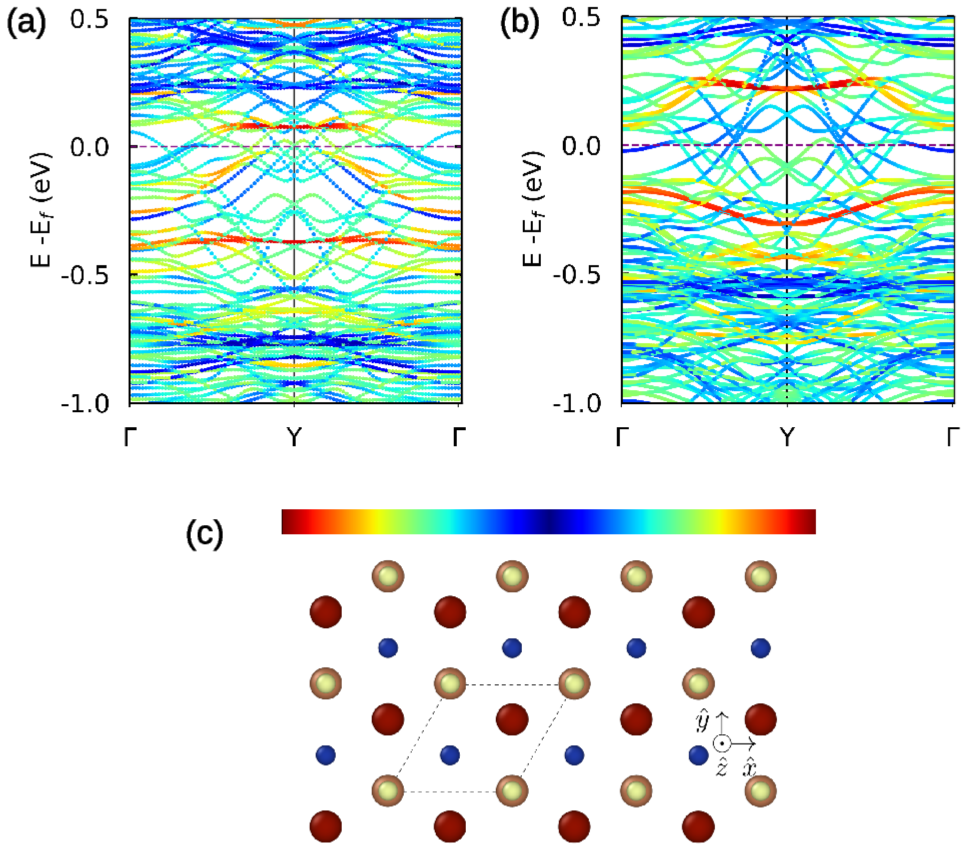

FIG. 2: Energy bands of armchair-edged nanoribbons made from single layers of (a) Fe_3GeTe_2 and (b) Fe_3GaTe_2. The horizontal dashed line indicates the the Fermi energy. The color-coding scheme (c) illustrates the spatial character of the corresponding eigenstates, with red denoting high probability amplitudes at the edge sites and blue indicating high probability amplitudes in the central region of the ribbon.

S3: ENERGY BANDS FOR ARMCHAIR NANORIBBONS

Fig. 2 presents the energy band structures of armchair-edged nanoribbons derived from Fe_3GeTe_2 and Fe_3GaTe_2 monolayers, as calculated using DFT. The color-coding scheme illustrates the spatial distribution of the corresponding eigenstates, with red indicating high-probability amplitudes at the edge sites and blue representing high probability amplitudes in the central region of the ribbon. Both ribbons have a width of approximately 17 Å. We observe two weakly dispersive (flat) bands localized at the edges—one above and one below the Fermi level. These flat bands have been linked to various intriguing physical phenomena, including superconductivity [19], the fractional quantum Hall effect [20], topological phases [21], and others [22].

S4: MAPPING THE ELECTRONIC HAMILTONIAN INTO THE SPIN HAMILTONIAN

The energy variation due to infinitesimal spin rotations at the sites i and j is given by

$$\delta\mathcal{H}_{ij} = -J_{ij}\delta\hat{m}_i\delta\hat{m}_j - \vec{D}_{ij}\cdot(\delta\hat{m}_i\times\delta\hat{m}_j) - \delta\hat{m}_i\mathcal{A}_{ij}\delta\hat{m}_j. \tag{4}$$

Using the Multiple Scattering Formalism (MSF) [23], and Lloyd's formula [24], we may write

$$\delta E_{ij} = -\frac{1}{\pi}\Im\int_{-\infty}^{\varepsilon_F} d\varepsilon Tr_{\sigma L}\left(\delta\mathbf{P}_i\tau_{ij}\delta\mathbf{P}_j\tau_{ji}\right), \tag{5}$$

where the trace is taken over spin σ and orbital angular momentum L indices of the *spd* basis. Here τ_{ij} represents the scattering path operator (SPO) and $\mathbf{P}_i$ is the inverse of the single site scattering operator (ISO). This equation describes only the leading term of the energy variation when the spins directions at sites i and j are rotated.

Following the steps of Refs. [25, 26] it is possible to rewrite

$$\begin{aligned}\delta E_{ij} = &-2\left(A_{ij}^{00} - \sum_{\mu=x,y,z} A_{ij}^{\mu\mu}\right)\delta\hat{m}_i\delta\hat{m}_j \\ &-2\sum_{\mu,\nu=x,y,z}\delta e_i^{\mu}\left(A_{ij}^{\mu\nu}+A_{ij}^{\nu\mu}\right)\delta e_j^{\nu} \\ &-2\left(\hat{A}_{ij}^{0\mu}-\hat{A}_{ij}^{\mu 0}\right)(\delta\hat{m}_i\times\delta\hat{m}_j)\ .\end{aligned} \tag{6}$$

where

$$A_{ij}^{\alpha\beta} = \frac{1}{4\pi}\Im\int_{-\infty}^{\varepsilon_F} d\varepsilon Tr_L\left(\delta_i G_{ij}^{\alpha}\delta_j G_{ji}^{\beta}\right) \tag{7}$$

and

$$\hat{A}_{ij}^{\alpha\beta} = \frac{1}{4\pi}\Re\int_{-\infty}^{\varepsilon_F} d\varepsilon Tr_L\left(\delta_i G_{ij}^{\alpha}\delta_j G_{ji}^{\beta}\right)\ , \tag{8}$$

where α and β run over 0, x, y and z.

Now, we can explicitly write the expressions for the magnetic parameters by comparing Eqs. 4 and 6 as

$$J_{ij} = A_{ij}^{00} - A_{ij}^{xx} - A_{ij}^{yy} - A_{ij}^{zz} \tag{9}$$

$$D^{\mu} = \hat{A}_{ij}^{0\mu} - \hat{A}_{ij}^{\mu 0} \tag{10}$$

$$\mathcal{A}_{ij} = A_{ij}^{\mu\nu} + A_{ij}^{\nu\mu} \tag{11}$$

The calculation based on Eqs.(9-11) are implemented in the RS-LMTO-ASA, but under the LMTO formalism. The connection between the MSF and the LMTO is not always trivial. In a general case, we have $p = \frac{C-E_v}{\Delta}$ and $T = \sqrt{\Delta}G\sqrt{\Delta}$, where C is the center of the band, E_v is the gravity center of the occupied bands, and G is the Green function. In the particular case of the work explored in this paper $p_iT_{ij}p_jT_{ji}$ assumes the form of $\frac{1}{4}\delta_iG_{ij}\delta_jG_{ji}$, where δ_i is the on-site exchange splitting and can be written as $\delta_i = \left(\mathcal{H}_{ii}^{\uparrow\uparrow} - \mathcal{H}_{ii}^{\downarrow\downarrow}\right)$. The inter-site Green's function can be calculated using either the Lanczos recursion procedure or the Chebyshev expansion algorithms, along with the kernel polynomial method. The choice of which recursion to use depends on the specific system under investigation. Some aspects of the Chebyshev and Lanczos recursion algorithms are discussed in Sec S5.

S5: EFFECTIVE SPIN INTERACTIONS

Figure 3 displays the calculated values of the effective exchange couplings between magnetic moments of Fe atoms separated by different interatomic distances in single layers of Fe_3GeTe_2 and Fe_3GaTe_2.

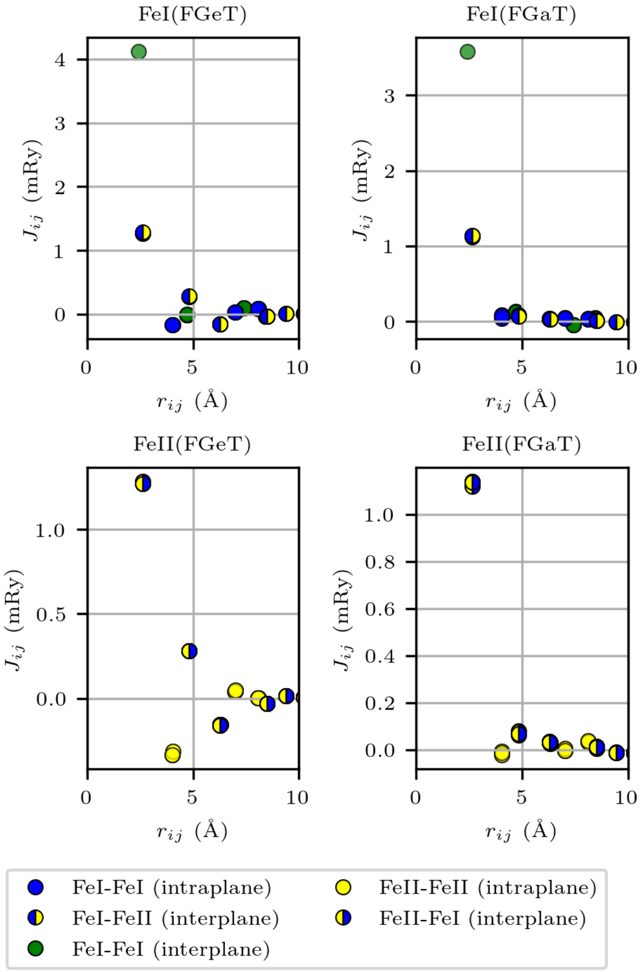

FIG. 3: Effective exchange couplings between magnetic moments of Fe atoms in single layers of Fe_3GeTe_2 and Fe_3GaTe_2, calculated as functions of their interatomic distances. Solid circles denote interactions between Fe atoms of the same type: (blue) Fe_I-Fe_I in-plane, (green) Fe_I-Fe_I inter-plane, (yellow) Fe_{II}-Fe_{II} in-plane. Inter-plane interactions between Fe atoms of distinct types are depicted by circles with two colors: (blue left/ yellow right) Fe_I-Fe_{II} and (yellow left/ blue right) Fe_{II}-Fe_I.

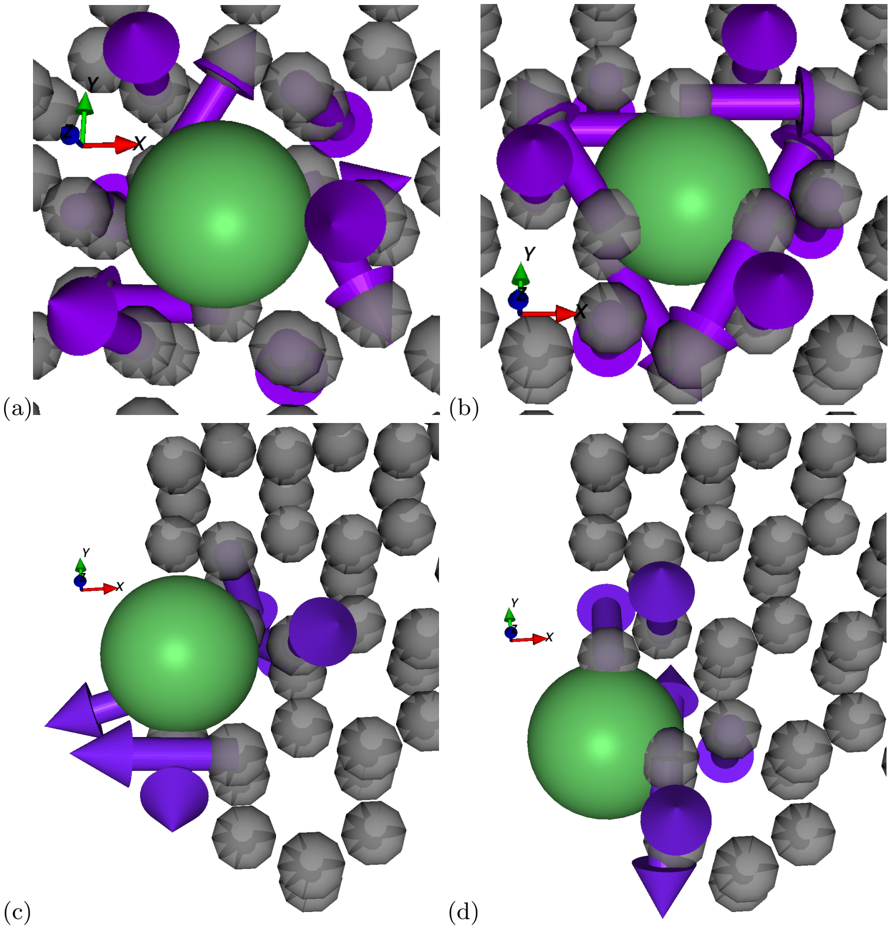

FIG. 4: DMI vector directions (indicated by purple arrows) between a Fe atom (green sphere) and its nearest neighboring Fe atoms (represented by gray spheres) in single layers of either Fe_3GeTe_2 or Fe_3GaTe_2. [a]. Panel (a) illustrates the directions of $\vec{D}_{ij}$ between Fe_I and its near neighbors, and panel (b) shows the directions between Fe_{II} and its near neighbors. Panel (c)/(d) depicts the $\vec{D}_{ij}$ directions between a Fe_I/(Fe_{II}) atom located at the edge of an armchair nanoribbon and its near neighbors.

[a] The directions of the $\vec{D}_{ij}$ vectors are the same for both materials, because they have the same lattice symmetry.

Figure 4 illustrates the directions of the vectors $\vec{D}_{ij}$ between neighboring Fe atoms in single layers of Fe_3GeTe_2 and Fe_3GaTe_2. In order to highlight the changes resulting from symmetry breaking in the transverse direction of the nanoribbons, panels (c) and (d) show the corresponding results for a Fe atom located at the edge of nanoribbons of the same materials. he Fe_I and Fe_{II}

S6: ATOMISTIC SPIN DYNAMICS AND SPIN ORBIT TORQUES

The atomistic spin dynamics simulations for the nanoribbons are performed using the UppASD code. The methodology is thoroughly explained in Ref [27], but in brief, we solve the Landau-Lifshitz-Gilbert (LLG) equation for localized atomic moments in the presence of an effective magnetic field given by $\mathbf{B}_i = \frac{\partial H}{\partial \hat{m}_i}$, where H is defined in Eq. 12.

$$H = -\sum_{i,j} J_{ij}\hat{m}_i\hat{m}_j - \sum_{i,j} \vec{D}_{ij} \cdot (\hat{m}_i \times \hat{m}_j) - \mathcal{K}\sum_{i} \left(\hat{m}_i \cdot \hat{m}_i^k\right)^2 \tag{12}$$

The site local effective field $\mathbf{B}_i$ enters the Landau-Lifshits-Gilbert equation, here expressed on the Landau-Lifshits form, which provides the equation of motion for the atomistic spins.

$$\dot{\mathbf{m}}_i = -\gamma'\mathbf{m}_i \times \mathbf{B}_i - \frac{\gamma'}{m_i}\mathbf{m}_i \times (\mathbf{m}_i \times \mathbf{B}_i) \tag{13}$$

where γ' is the renormalized gyromagnetic ratio $\gamma' = \frac{\gamma}{1+\alpha^2}$ and α is the Gilbert damping.

The presence of currents in the sample can cause spin torques from both regular spin transfer torque (STT) as well as spin-orbit torques (SOT). In an atomistic spin dynamics description, the SOT contribution $\mathbf{B}_{SOT}$ to the effective magnetic field $\mathbf{B}_i$ on magnetic moment $\mathbf{m}_i$ can be expressed as[28]

$$\mathbf{B}_i^{\mathrm{SOT}} = B_i^{\mathrm{P}}(\boldsymbol{\sigma} - \alpha\,\mathbf{m}_i \times \boldsymbol{\sigma}) \quad + B_i^{\mathrm{R}}(\mathbf{m}_i \times \boldsymbol{\sigma} + \alpha\,\boldsymbol{\sigma}) \tag{14}$$

where $\boldsymbol{\sigma}$ is the spin polarisation unit vector while $\mathbf{B}_i^{\mathrm{P}}$ and $\mathbf{B}_i^{\mathrm{R}}$ denote the field strength prefactors for the precessional (P) and relaxational (R) contributions to the effective field. These prefactors are given as[28]

$$B_i^{\mathrm{P}} = \frac{\hbar\alpha^2}{2e\mu_s} j\theta_{\mathrm{SH}} \tag{15}$$

$$B_i^{\mathrm{R}} = \beta B_i^{\mathrm{P}} = \beta_i \frac{\hbar\alpha^2}{2e\mu_s} j\theta_{\mathrm{SH}}. \tag{16}$$

The relative importance between the precession and relaxation contributions to the SOT can not be determined from first-principles and is instead defined by the empirical scaling parameter β. While β can be larger than unity it is often expected to be small and in this work we used $\beta = \alpha$.

The material specific spin Hall angle θ_{SH} can be calculated from electronic structure methods (see eg. Ref. [29]) but in this work we have used the following experimental estimates of the spin Hall angle for Mn_3Sn, $\theta_{\mathrm{SH}} = 0.3$, emulating a situation where the nanoribbons are deposited on Mn_3Sn.

S7: THE RECURSION METHOD

We are interested in computing the spectral function $A(\omega)$ for systems where we only are able to apply the system Hamiltonian $\hat{H}$ to a single state vector $|\psi\rangle$

$$|\psi'\rangle = \hat{H}|\psi\rangle \,, \tag{17}$$

combined with the ability to compute state vector overlaps $\langle\psi'|\psi\rangle$. Formally the spectral function is given by

$$A_{ab}(\omega) = \sum_{\Gamma} \langle\Psi_0|c_a|\psi_\Gamma\rangle\langle\psi_\Gamma|c_b^\dagger|\Psi_0\rangle\delta(\omega + E_0 - E_\Gamma) \tag{18}$$

where ψ_Γ and E_Γ are eigenvectors and eigenstates of $\hat{H}$ and Ψ_0 and E_0 the groundstate and groundstate energy, respectively.

Lanczos recursion

The Lanczos method computes the spectral function in terms of the Green's function

$$A_{ab}(\omega) = -\frac{1}{\pi}\lim_{\delta\to 0} \mathrm{Im}\left[G_{ab}(\omega + i\delta)\right] \tag{19}$$

where

$$G_{ab}(z) = \langle\Psi_0|c_a \frac{1}{z + E_0 - \hat{H}} c_b^\dagger|\Psi_0\rangle \tag{20}$$

For the diagonal Green's function $G_{aa}(z)$ the Laczos recursion [30, 31] iteratively builds up a tri-diagonal representation using the initial relations

$$|\psi_{-1}\rangle = 0\,, \quad |\psi_0\rangle = c_a^\dagger|\Psi_0\rangle/\beta_0\,, \quad \beta_0 = ||c_a^\dagger|\Psi_0\rangle|| \tag{21}$$

and the recursion

$$|\psi'\rangle = \hat{H}|\psi_n\rangle - \beta_n|\psi_{n-1}\rangle\,, \qquad \alpha_n = \langle\psi_n|\psi'\rangle\,, \tag{22}$$

$$|\psi''\rangle = |\psi'\rangle - \alpha_n|\psi_n\rangle\,, \qquad \beta_{n+1} = ||\psi''||\,, \tag{23}$$

$$|\psi_{n+1}\rangle = |\psi''\rangle/\beta_{n+1}\,. \tag{24}$$

The real frequency Green's function $G_{nn}(z)$ can then be obtained from the from continued fraction

$$G_{aa}(z) = \cfrac{\beta_0^2}{z - \alpha_0 - \cfrac{\beta_1^2}{z - \alpha_1 - \cfrac{\beta_2^2}{z - \alpha_2 - \ldots}}}\,, \tag{25}$$

giving the spectral function $A_{aa}(\omega)$ using Eq. (19), for more details see Ref. [32].

Off-diagonal components $G_{ab}(\omega)$, with $a \neq b$, can readily be computed using two auxilliary response functions [31], $G_{ab}(z) = \frac{1}{2}\left(G_{++}(z) - G_{--}(z)\right)$ where

$$G_{\pm\pm}(z) = \langle\Psi_0|c_\pm \frac{1}{z + E_0 - \hat{H}} c_\pm^\dagger|\Psi_0\rangle \tag{26}$$

and $c_\pm = (c_a \pm c_b)/\sqrt{2}$.

The Lanczos method excels at approximating the moments of the spectral function. However, since the recursion tends to loose orthogonality it rapidly becomes unstable when performing a large number of recursions [33]. Therefore it is not possible to use Lanczos recursion to compute the spectral function to high accuracy.

Chebyshev recursion

The Chebyshev recursion is a numerically robust alterantive where the recursion is stable even after thousands or more recursions. However, the recursion requires that the Hamiltonian operator $\tilde{H}$ has spectral support on $(-1, 1)$.

Thus, given a generic Hamiltonian $\hat{H}$ its extremal eigenvalues E_{min} and E_{max} determines the scaling and shift,

$$W = \frac{E_{max} - E_{min}}{2 - \epsilon}\,, \quad \bar{E} = \frac{E_{max} + E_{min}}{2}\,, \tag{27}$$

required for the Hamiltonian

$$\tilde{H} = \frac{1}{W}\left(H - \bar{E}\mathbf{1}\right), \tag{28}$$

to have an eigenvalues $\tilde{E} \in (-1, 1)$, excluding the bounaries as $0 < \epsilon \ll 2$.

The Chebyshev recursion uses the starting point

$$|\psi_0\rangle = c_a^\dagger |\Psi_0\rangle\,, \qquad \mu_0 = \langle\psi_0|\psi_0\rangle\,, \tag{29}$$

$$|\psi_1\rangle = \tilde{H}|\psi_0\rangle\,, \qquad \mu_1 = \langle\psi_1|\psi_0\rangle\,, \tag{30}$$

and recursion relations

$$|\psi_{n+1}\rangle = 2\,\tilde{H}\,|\psi_n\rangle - |\psi_{n-1}\rangle\,, \tag{31}$$

$$\mu_{2n+1} = 2\langle\psi_{n+1}|\psi_n\rangle - \mu_1\,, \tag{32}$$

$$\mu_{2n+2} = 2\langle\psi_{n+1}|\psi_{n+1}\rangle - \mu_0\,, \tag{33}$$

giving the Chebyshev moments μ_n.

For a given finite number N of exact moments μ_n the best approximation of the spectral function is given by

$$A_{aa}(\omega) = \frac{1}{\pi a}\frac{1}{\sqrt{1-x^2}}\left[\tilde{\mu}_0 + 2\sum_{n=1}^{N}\tilde{\mu}_n T_n(x)\right], \tag{34}$$

where $x = (\omega - \bar{E})/W$, $T_n(x)$ is the n:th order Chebyshev polynomial, and $\tilde{\mu}_n = g_n \mu_n$ where g_n is the coefficients of the Jackson kernel [34], for details see Ref. [33].

S8: ADDITIONAL SIMULATION DATA

Critical temperatures

The simulated critical temperatures for the considered systems can be seen in Table II. They have been determined by performing thermodynamical sampling of ASD simulations and then identifying the maximum of the magnetic specific heat.

Temperature dependence of switching dynamics

In Figs. 5 and 6, we show how the switching dynamics of armchair nano-ribbons of Fe_3GeTe_2 and Fe_3GaTe_2 is affected by temperature. Increasing the temperature results in faster switching. However, close or above the critical temperature, there is no proper switching since the net magnetization is close to zero at $t = 0$, i.e. the systems are effectively paramagnetic. Then the resulting torque from the current driven SOT results in a finite net magnetization along the direction imposed by the SOT. Relevant working temperatures would thus be at T=100K and below for these systems.

System	Geometry	T_C (K)
Fe_3GeTe_2	Monolayer	280
	Armchair	240
	Bulk Armchair	280
	Zigzag	380
Fe_3GaTe_2	Monolayer	420
	Armchair	310
	Bulk Armchair	460

TABLE II: Simulated critical temperatures of Fe_3GeTe_2 and Fe_3GaTe_2 nano-ribbons and monolayers.

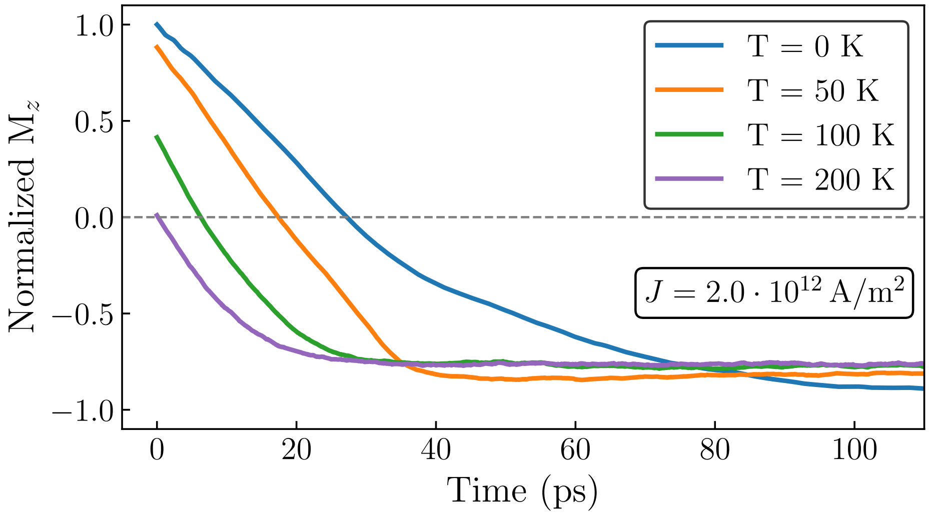

FIG. 5: Temperature dependence switching dynamics for Fe_3GeTe_2

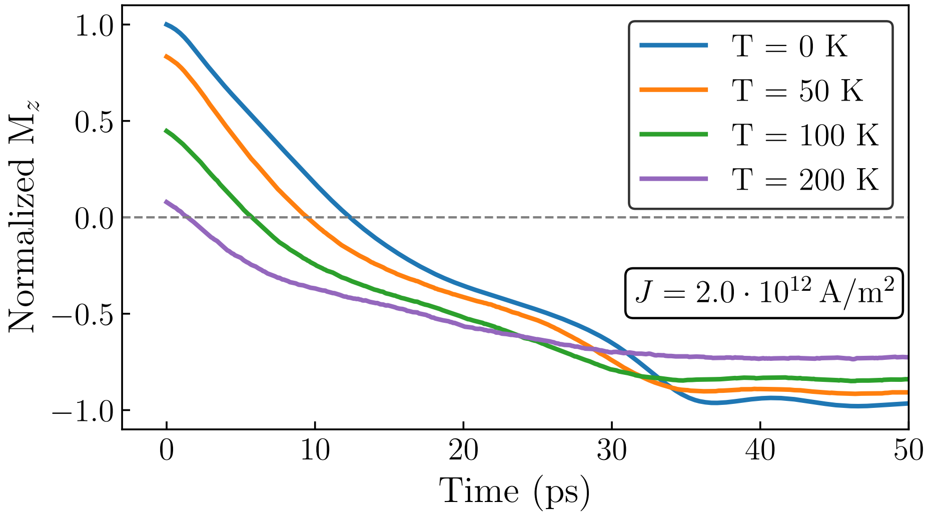

FIG. 6: Temperature dependence switching dynamics for Fe_3GaTe_2

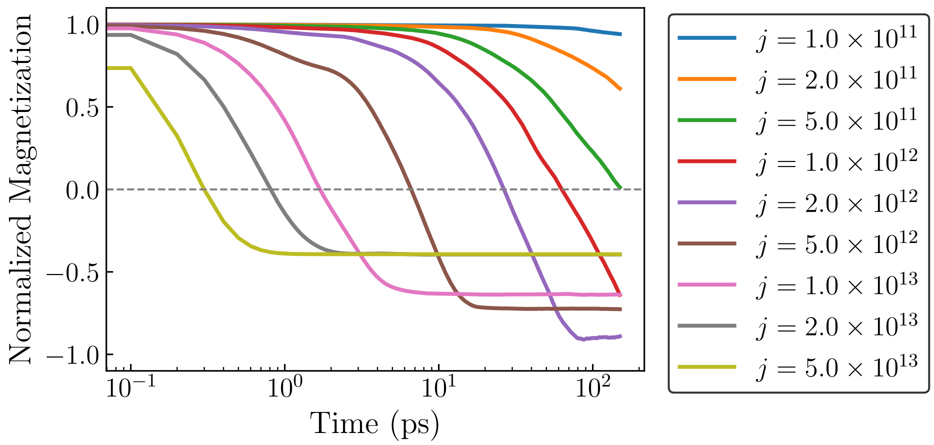

FIG. 7: Current dependence switching dynamics for Fe_3GeTe_2 armchair nanoribbon

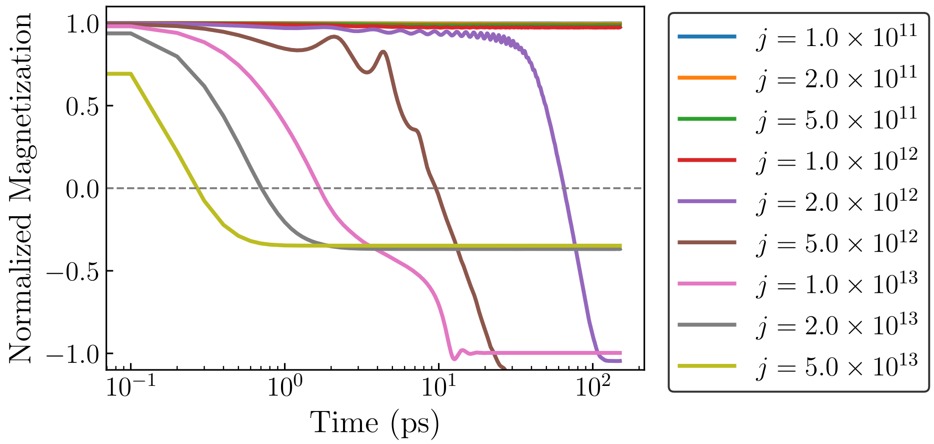

FIG. 8: Current dependence switching dynamics for Fe_3GeTe_2 zigzag nanoribbon

Current dependence of switching dynamics

Figs. 7, 8, and 9 show how the current density affects the current driven switching for Fe_3GeTe_2 and Fe_3GaTe_2 nano-ribbons. Here we have considered armchair and zigzag configurations for Fe_3GeTe_2 and armchair for Fe_3GaTe_2. In these figures, the current is present throughout the full simulation, in contrast to Fig. 4 in the main text. As expected, switching times decrease with increasing current densities. Current densities lower than $j = 1.0 \times 10^{1}1$ do not result in any switching dynamics within the simulation time, indicating that the SOT is not able to overcome the anisotropy field of the nano-ribbons. Looking at the end of the different switching scenarios, it can be noticed that for lower current densities, a typical switching behaviour of the m_z component of the magnetization occurs. On the other hand, larger current densities result in a less pronounced switching by looking at the m_z component of the magnetization, which is the component shown in Figs. 7- 9. This is because as the current density increases, the resulting SOT overcomes the uniaxial anisotropy of the nano-ribbons and from the symmetry of the SOT, the resulting magnetization obtains a large m_y component (now shown). Thus, the total magnetization of the samples is in fact the same at the end of the switching as at the start.

Switching dynamics for bulk-like exchange interactions

In order to emphasize the effect of the non-collinear magnetism present at the edges of the nano-ribbons, we also performed switching simulations for hypothetical systems consisting of the same armchair nano-ribbon geometry as

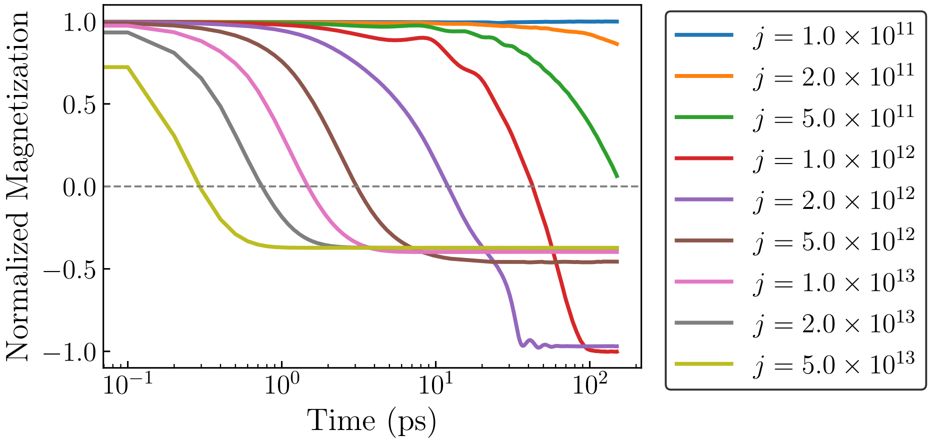

FIG. 9: Temperature dependence switching dynamics for Fe_3GaTe_2 armchair nanoribbon

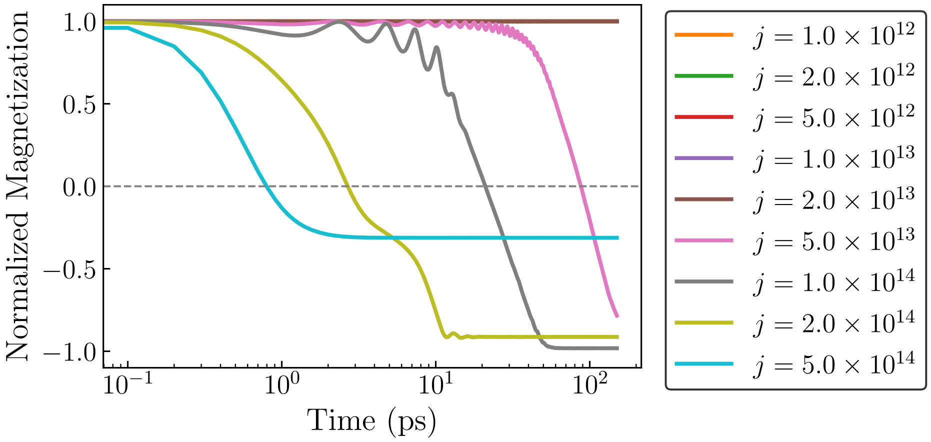

FIG. 10: Current dependence switching dynamics for Fe_3GeTe_2 armchair nanoribbon using exchange interactions for a pristine monolayer.

considered above, but with the important distinction that the exchange interactions included in the simulations are not the ones that have been calculated for the finite nano-ribbons. Instead we here use the exchange interactions calculated for the periodic monolayers of Fe_3GeTe_2 and Fe_3GaTe_2 which are both shown to order ferromagnetic. With these monolayer interactions, the resulting magnetic order of the hypothetical nano-ribbons is ferromagnetic as well. Hence, comparing the switching dynamics for these hypothetical "bulk-like" nano-ribbons with the proper finite systems indicates the effect of the non-collinear edges present in the proper nano-ribbons. The switching scenarios for the proper Fe_3GeTe_2 and Fe_3GaTe_2 nano-ribbons were shown in Figs. 7 and 9 and in Figs. 10 and 11 we show the switching dynamics of the hypothetical ferromagnetic nano-ribbons. Comparing the dynamics, we see that for Fe_3GeTe_2, almost 50x larger current densities are needed to switch the ferromagnetic armchair ribbon on the same time scale as the non-collinear armchair ribbon, clearly showcasing the effect of the non-collinear edges. For Fe_3GaTe_2, the effect is not as extreme but still a 5x larger current density is needed here.

* ramon.cardias@cbpf.br

[1] J. P. Perdew, K. Burke, and M. Ernzerhof, Generalized gradient approximation made simple, Phys. Rev. Lett. **77**, 3865 (1996).
[2] G. Kresse and D. Joubert, From ultrasoft pseudopotentials to the projector augmented-wave method, Phys. Rev. B **59**, 1758 (1999).

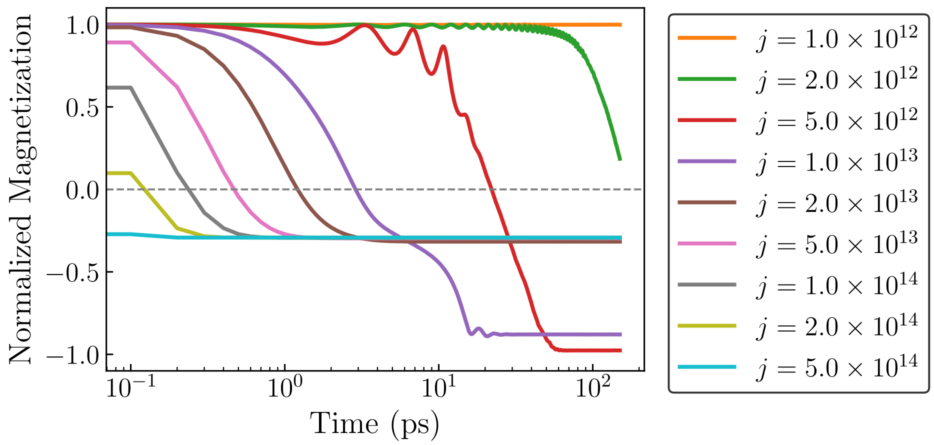

FIG. 11: Temperature dependence switching dynamics for Fe_3GaTe_2 armchair nanoribbon using exchange interactions for a pristine monolayer.

[3] A. D. Corso, Pseudopotentials periodic table: From h to pu, Comput. Mater. Sci. **95**, 337 (2014).

[4] M. Costa, N. M. R. Peres, J. Fernández-Rossier, and A. T. Costa, Nonreciprocal magnons in a two-dimensional crystal with out-of-plane magnetization, Phys. Rev. B **102**, 014450 (2020).

[5] A. M. Ruiz, D. L. Esteras, D. López-Alcalá, and J. J. Baldoví, On the Origin of the Above-Room-Temperature Magnetism in the 2D van der Waals Ferromagnet Fe3GaTe2, Nano Letters **24**, 7886 (2024), publisher: American Chemical Society.

[6] L. A. Agapito, A. Ferretti, A. Calzolari, S. Curtarolo, and M. Buongiorno Nardelli, Effective and accurate representation of extended bloch states on finite hilbert spaces, Phys. Rev. B **88**, 165127 (2013).

[7] L. A. Agapito, M. Fornari, D. Ceresoli, A. Ferretti, S. Curtarolo, and M. Buongiorno Nardelli, Accurate tight-binding hamiltonians for two-dimensional and layered materials, Phys. Rev. B **93**, 125137 (2016).

[8] F. T. Cerasoli, A. R. Supka, A. Jayaraj, M. Costa, I. Siloi, J. Sławińska, S. Curtarolo, M. Fornari, D. Ceresoli, and M. Buongiorno Nardelli, Advanced modeling of materials with paoflow 2.0: New features and software design, Computational Materials Science **200**, 110828 (2021).

[9] M. Costa, G. R. Schleder, M. Buongiorno Nardelli, C. Lewenkopf, and A. Fazzio, Toward realistic amorphous topological insulators, Nano Letters **19**, 8941 (2019).

[10] A. T. Costa, M. Costa, and J. Fernández-Rossier, Ising and xy paramagnons in two-dimensional $2h-$nbse$_2$, Phys. Rev. B **105**, 224412 (2022).

[11] H. Kontani, T. Tanaka, D. S. Hirashima, K. Yamada, and J. Inoue, Giant orbital hall effect in transition metals: Origin of large spin and anomalous hall effects, Phys. Rev. Lett. **102**, 016601 (2009).

[12] T. Tanaka, H. Kontani, M. Naito, T. Naito, D. S. Hirashima, K. Yamada, and J. Inoue, Intrinsic spin hall effect and orbital hall effect in $4d$ and $5d$ transition metals, Phys. Rev. B **77**, 165117 (2008).

[13] D. Go, D. Jo, C. Kim, and H.-W. Lee, Intrinsic spin and orbital hall effects from orbital texture, Phys. Rev. Lett. **121**, 086602 (2018).

[14] D. Jo, D. Go, G.-M. Choi, and H.-W. Lee, Spintronics meets orbitronics: Emergence of orbital angular momentum in solids, npj Spintronics **2**, 19 (2024).

[15] S. Bhowal and G. Vignale, Orbital hall effect as an alternative to valley hall effect in gapped graphene, Phys. Rev. B **103**, 195309 (2021).

[16] T. P. Cysne, S. Bhowal, G. Vignale, and T. G. Rappoport, Orbital hall effect in bilayer transition metal dichalcogenides: From the intra-atomic approximation to the bloch states orbital magnetic moment approach, Phys. Rev. B **105**, 195421 (2022).

[17] D. Xiao, J. Shi, and Q. Niu, Berry phase correction to electron density of states in solids, Phys. Rev. Lett. **95**, 137204 (2005).

[18] D. Kim, C. Lee, B. G. Jang, K. Kim, and J. H. Shim, Drastic change of magnetic anisotropy in fe3gete2 and fe4gete2 monolayers under electric field studied by density functional theory, Scientific Reports **11**, 17567 (2021).

[19] Y. Cao, V. Fatemi, S. Fang, K. Watanabe, T. Taniguchi, E. Kaxiras, and P. Jarillo-Herrero, Unconventional superconductivity in magic-angle graphene superlattices, Nature **556**, 43 (2018).

[20] E. Tang, J.-W. Mei, and X.-G. Wen, High-temperature fractional quantum hall states, Phys. Rev. Lett. **106**, 236802 (2011).

[21] K. Sun, Z. Gu, H. Katsura, and S. Das Sarma, Nearly flatbands with nontrivial topology, Phys. Rev. Lett. **106**, 236803 (2011).

[22] J. G. Checkelsky, B. A. Bernevig, P. Coleman, Q. Si, and S. Paschen, Flat bands, strange metals and the kondo effect, Nature Reviews Materials **9**, 509 (2024).

[23] H. Ehrenreich and L. Schwartz, The electronic structure of alloys**research supported by the national science foundation under grant no. dmr-72–02977 a03, dmr 72–03020 a05, and dmr 27–03209 a01 (Academic Press, 1976) pp. 149–286.

[24] P. Lloyd and P. Smith, Multiple scattering theory in condensed materials, Advances in Physics **21**, 69 (1972), https://doi.org/10.1080/00018737200101268.

[25] A. Szilva, M. Costa, A. Bergman, L. Szunyogh, L. Nordström, and O. Eriksson, Interatomic exchange interactions for finite-temperature magnetism and nonequilibrium spin dynamics, Phys. Rev. Lett. **111**, 127204 (2013).

[26] R. Cardias, A. Szilva, M. M. Bezerra-Neto, M. S. Ribeiro, A. Bergman, Y. O. Kvashnin, J. Fransson, A. B. Klautau, O. Eriksson, and L. Nordström, First-principles dzyaloshinskii–moriya interaction in a non-collinear framework, Scientific Reports **10**, 20339 (2020).

[27] O. Eriksson, A. Bergman, L. Bergqvist, and a. J. Hellsvik, *Atomistic Spin Dynamics: Foundations and Applications* (Oxford University Press, Oxford, New York, 2017).

[28] A. Meo, C. E. Cronshaw, S. Jenkins, A. Lees, and R. F. L. Evans, enSpin-transfer and spin-orbit torques in the Landau–Lifshitz–Gilbert equation, Journal of Physics: Condensed Matter **35**, 025801 (2022), publisher: IOP Publishing.

[29] M. Gradhand, D. V. Fedorov, P. Zahn, and I. Mertig, Spin hall angle versus spin diffusion length: Tailored by impurities, Phys. Rev. B **81**, 245109 (2010).

[30] R. Haydock, V. Heine, and M. J. Kelly, Electronic structure based on the local atomic environment for tight-binding bands, Journal of Physics C: Solid State Physics **5**, 2845 (1972).

[31] R. Haydock, V. Heine, and M. J. Kelly, Electronic structure based on the local atomic environment for tight-binding bands. ii, Journal of Physics C: Solid State Physics **8**, 2591 (1975).

[32] E. Dagotto, Correlated electrons in high-temperature superconductors, Rev. Mod. Phys. **66**, 763 (1994).

[33] A. Weiße, G. Wellein, A. Alvermann, and H. Fehske, The kernel polynomial method, Rev. Mod. Phys. **78**, 275 (2006).

[34] T. J. Rivlin, *An Introduction to the Approximation of Functions* (Dover Publications Inc., 180 Varick Street, New York, NY 10014, 1981).
